# Association of CALLY index and CLR with COPD risk in middle-aged and older Americans: evidence from NHANES 2017–2020

**DOI:** 10.3389/fmed.2025.1535415

**Published:** 2025-04-17

**Authors:** Jiaji Zhou, Wenyi Du, Hanzhou Huang, Yongqi Chen, Leyan Chen, Mingfeng Zheng

**Affiliations:** ^1^Department of Thoracic Surgery, The Affiliated Wuxi People’s Hospital of Nanjing Medical University, Wuxi, China; ^2^Wuxi Medical Center, Affiliated With Nanjing Medical University, Wuxi, China; ^3^Department of General Surgery, The Affiliated Wuxi People’s Hospital of Nanjing Medical University, Wuxi, China

**Keywords:** inflammation indicators, chronic obstructive pulmonary disease, NHANES, CALLY index, CLR

## Abstract

**Background:**

Chronic obstructive pulmonary disease (COPD) is marked by restrictions on airflow, leading to a gradual and irreversible reduction in lung function. This study assessed the predictive value of hematological inflammatory biomarkers, specifically the C-reactive protein-albumin-lymphocyte (CALLY) index and the C-reactive protein to lymphocyte ratio (CLR), for determining COPD risk in United States adults aged 40 and above.

**Methods:**

Data were sourced from the National Health and Nutrition Examination Survey (NHANES) covering the period from 2017 to March 2020. The relationship between inflammatory markers, including the CALLY index, CLR, and their components, and COPD was assessed using multivariate logistic regression. Subgroup analyses explored the relationship between the CALLY index, CLR, and COPD, while restricted cubic spline (RCS) analyses evaluated potential non-linearity. The predictive performance of these biomarkers for COPD risk was assessed using receiver operating characteristic (ROC) curve analysis.

**Results:**

After controlling for confounders, for every one-unit increase in the CALLY index (converted to natural logarithm), the prevalence of COPD decreased by 19% (OR = 0.81, 95% CI: 0.71–0.92, *P* = 0.001). Conversely, for every one-unit increase in the CLR (converted to natural logarithm), the prevalence of COPD increased by 23% (OR = 1.23, 95% CI: 1.08–1.40, *P* < 0.001). The linear negative correlation between the CALLY index and COPD was demonstrated by using RCS curves, while the CLR exhibited a positive association. After being fully adjusted, both the CALLY index and the CLR yielded an adjusted area under the curve (AUC) of 0.831 for predicting the risk of COPD, demonstrating excellent predictive capability.

**Conclusion:**

The study identifies a linear negative relationship between the CALLY index and COPD, unaffected by potential confounders. A higher CLR is linked to an elevated risk of COPD development. Both the CALLY index and CLR were superior in predicting the risk of developing COPD. Our findings emphasize that the CALLY index and CLR may be a new inflammatory early warning biomarker for COPD.

## 1 Introduction

The primary classifications of chronic respiratory disease (CRPD) encompass COPD and asthma (1). The condition is primarily characterized by airflow limitation (AL). The global incidence of CRPD is notably high and demonstrates an upward trend annually (1, 2). CRPD prevalence and severity are silent plagues that erode society’s health (3). In 2019, there were more than 400 million CRPD cases and more than 4 million deaths globally, an increase of 28.5 % and 39.8 % from 1990, respectively (3). Deaths from COPD, the leading cause of mortality among CRPDs, surpassed 3 million. In addition, there are more than 200 million cases of asthma, which has the highest prevalence among CRPD (3). All of this indicates the current grim situation of CRPD. Therefore, screening for CRPD at an early stage, before it becomes symptomatic and progresses, is crucial.

At an early stage of CRPD, lung function assessment has advantages (4–6). However, lung function is only tested at the onset of respiratory symptoms. Furthermore, lung function testing is not widely used as part of health screening or primary care (7, 8). A United States study indicates that despite improvements in spirometer use for COPD detection and management in primary care following the National Lung Health Education Program (NLHEP), 70% of COPD patients remain undiagnosed through spirometry (9). In epidemiology, a straightforward biomarker for early-stage lung dysfunction could aid in the disease’s assessment, management, and treatment.

Inflammation holds a vital position in lung dysfunction, with various biomarkers such as C-reactive protein (CRP), lymphocytes, neutrophil-to-lymphocyte ratio (NLR), and platelets being indicative of its severity and progression. These markers are cost-effective, stable, and reproducible (10, 11). A relatively new nutritional indicator of inflammation is the CALLY index. The CALLY index was initially identified as a quick and straightforward biomarker for evaluating the anticipated progression of patients with non-small cell lung cancer (12). CALLY is a novel, comprehensive inflammatory index that integrates serum albumin, lymphocyte count, and CRP. These interrelated components serve as indicators of an individual’s nutritional status, immune function, and level of inflammation, respectively (13). The CALLY score’s clinically accessible parameters have garnered increasing attention. CLR, a composite biomarker combining neutrophils and CRP, is also a cost-effective and accessible option. CLR is linked to poor prognosis in non-small cell patients, aiding in predicting postoperative outcomes (14).

Currently, there is a lack of academic findings on the relationship between two inflammatory markers, the CALLY index and CLR, and COPD. In the past, most of the research on the CALLY index and CLR in pulmonary diseases was confined to lung cancer (12, 14). There were few studies on COPD, which is closely associated with inflammation. Recently, the team led by Yu Ding explored the influence of the CALLY index on the survival prognosis of COPD patients (15). However, the database used in this study had relatively old data and only focused on this single CALLY index. In contrast, the data our team used in this study is more recent and can better represent the current clinical situation. Moreover, we combined multiple inflammatory indicators to compare their impacts on COPD patients comprehensively and investigated the predictive performance among them. This study was like a detective seeking to evaluate the connection between blood inflammatory biomarkers and the risk of COPD and to explore whether these biomarkers could act as early warning beacons for COPD using data from the 2017 to March 2020 NHANES in the United States.

In addition, existing studies have shown that inflammatory markers are of great significance in cardiovascular diseases and other aspects. For example, the research team led by Karakayali et al. (16) found that the systemic immune-inflammation index (SII), as a new inflammatory marker, is associated with coronary artery disease. Similarly, the C-reactive protein to albumin ratio (CAR) has also been proven to be related to the severity of coronary artery disease (17). These studies suggest that inflammatory markers may play a crucial role in various diseases and also provide a reference for exploring the relationship between the CALLY index, CLR, and COPD.

## 2 Materials and methods

### 2.1 Data source

**FIGURE 1:**
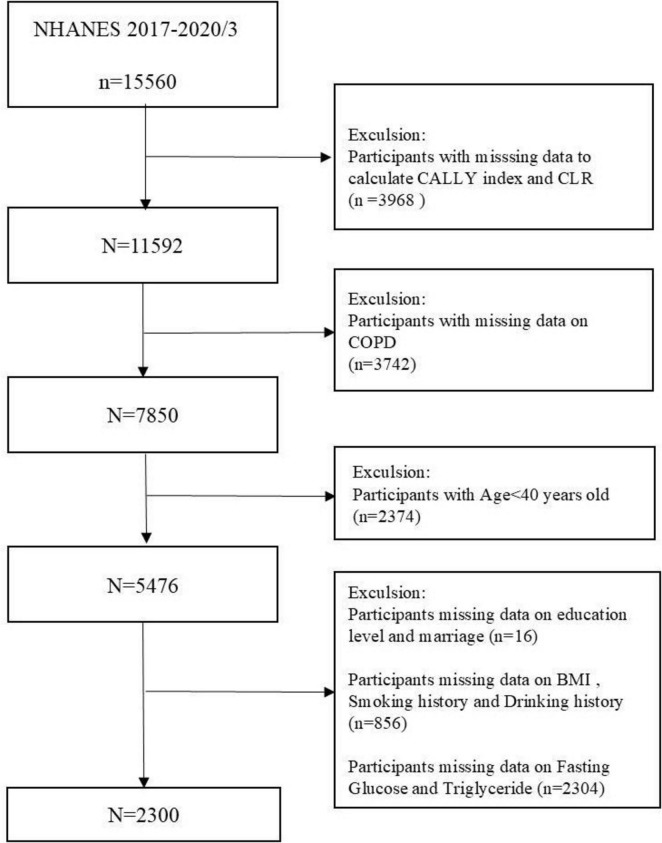
Flowchart of participant selection.

This survey used NHANES data from 2017 to 2020 in March, and 15,560 participants took part in the 2017–2020/3 survey cycle. We employed rigorous inclusion and exclusion criteria to guarantee comprehensive and accurate results. Participants younger than 40 years (*n* = 2374) and those lacking data for lymphocyte (*n* = 3409), HS C-reactive protein (CRP) (*n* = 553), and albumin (*n* = 6) were excluded. Subjects with incomplete COPD data (*n* = 3,742) and those missing data on at least one covariate—education, marital status, body mass index (BMI), alcohol use, smoking status, fasting glucose (FBG), or triglycerides (TG) (*n* = 3,176)—were excluded. Figure 1 provides a flowchart that clarifies the screening process. Participants in the NHANES study gave informed written consent, and the National Center for Health Statistics (NCHS) validated all data before public release.

### 2.2 Measurement of white blood cell, monocyte, C-reactive protein, NLR, CALLY index and CLR

High sensitivity C-reactive protein (HS-CRP) was selected for use in this study due to its high sensitivity characteristics and was quantified in mg/L. The high-sensitivity near-infrared particle immunoassay rate was adopted for experimental operations. For detailed methods, please visit: (HS-CRP Labor)^1^. The NHANES MEC routinely conducted complete blood count (CBC) tests using the Beckman Coulter method, providing data on red cell distribution width, as well as erythrocyte and platelet counts. CALLY index, CLR, and NLR were obtained using CBC information from the NHANES database. For comprehensive details on lab techniques, quality control, and data handling, visit the NHANES website at https://wwwn.cdc.gov/Nchs/Nhanes/2013-2014/CBC_H.htm.

The neutrophil-to-lymphocyte ratio (NLR) for each participant was determined by calculating the quotient of the absolute neutrophil count and the absolute lymphocyte count. Studies have been conducted to confirm the potential of this indicator of inflammation in predicting the risk of death in COPD (18). This study investigates the ability of the CALLY index and CLR, two novel inflammation indicators, to predict the risk of COPD compared to inflammation indicators such as NLR and its related components. The same blood sample collected from each patient was used to calculate the CALLY index and CLR with the following formula: the CALLY index is calculated by multiplying albumin (g/L) with lymphocyte count (10^9^/L) and dividing the product by the CRP level (mg/L) multiplied by 10. CLR is obtained by dividing CRP (mg/L) by lymphocyte count (10^9^/L).

Each individual who has been incorporated underwent routine hematological and biochemical measurements. Metabolic indicators, including FBG and TG, were acquired via standard biochemical analysis, encompassing detailed sample collection and laboratory testing. Detailed sample collection and laboratory testing methodologies can be found in the Laboratory/Medical Technician Procedures Manual on the NHANES website.

### 2.3 Dependent variables and covariates

Participants were categorized as having COPD in the event that they affirmed any one of the following circumstances: being diagnosed with COPD, chronic bronchitis, or emphysema by a medical practitioner. Numerous earlier studies using NHANES data have effectively employed this approach to identify COPD patients (19).

To enhance the study’s comprehensiveness, additional variables were incorporated, informed by prior research findings and the clinical team’s collective expertise. The NHANES demographic dataset encompasses variables including gender, age (categorized as 40–60 years and over 60 years), race, and education. Data on smoking history (at least 100 cigarettes in a lifetime), drinking history (at least 4/5 or more drinks every day), diabetes, and high blood pressure were obtained from the questionnaire data on the NHANES website. In the NHANES survey, BMI was assessed by trained health technicians at the Mobile Examination Centre (MEC) and classified into three categories: below average or healthy (< 25.0 kg/m^2^), overweight (25.0–30.0 kg/m^2^), and obese (= 30 kg/m^2^). Educational attainment was divided into five categories according to the NHANES website. For details, please refer to: (see text footnote 1). Married, cohabitating, and widowed individuals are classified as the first category; divorced, separated, or widowed individuals are classified as the second category; and unmarried individuals are classified as the third category. Please visit the NHANES website for more information about these covariates.

### 2.4 Statistical analysis

The median and interquartile range assessed continuous variables. Variables of a categorical nature were described using frequency and percentage. Categorical variables were analyzed using the chi-square test or Fisher’s exact test if the expected frequency was less than five. The Wilcoxon rank-sum test was employed to evaluate differences in continuous variables across groups. Log transformations were applied to enhance data accuracy prior to conducting statistical analyses on the CALLY index and CLR entry groups. The relationship between the study variables and COPD was analyzed using logistic regression models. Model 1 was unadjusted and did not include any covariates. Model 2 included key demographic variables, including age, race, and gender. Model 3 optimizes Model 2 by incorporating multiple covariates such as education level, marital status, smoking history, asthma, and FBG on the basis of Model 2. The comprehensively adjusted model was used to investigate possible layered connections between the study variables and COPD. Subgroup analyses examined the interactions between COPD and variables like gender, race, lifestyle habits, and comorbidities. RCS smoothing curve fitting was employed to assess the association between the two variables. Multivariate analysis models were adjusted based on significant variables identified in Supplementary Table 1. The predictive capabilities of the CALLY index, CLR, NLR, white blood cells, monocytes, and albumin for COPD risk were evaluated using univariate and multivariate ROC analyses, with results expressed as the area under the ROC curve (AUC). A two-sided *p*-value below 0.05 was deemed significant.

These statistical methods allow a comprehensive analysis of the possible links between the CALLY index and CLR, two novel indicators of inflammation, and COPD. The data was analyzed using R (version 4.2.1) and MSTATA (v0.92). Statistical significance is determined by 0.05 or less as a *P*-value.

## 3 Results

### 3.1 Description of the characteristics of subjects

In Table 1, information is provided on the demographics, examinations, laboratory tests, and questionnaires of participants in the 2017–2020/3 NHANES survey. The study included 2,300 participants, with 1,207 men and 1,093 women, divided into “COPD patients” and “non-COPD patients” groups based on their COPD status. The analysis revealed significant disparities among the groups concerning demographic characteristics, lifestyle factors, and comorbidities. The findings suggest that regular smoking elevates the likelihood of developing COPD. Individuals diagnosed with COPD tend to be older, predominantly female, and more frequently identify as non-Hispanic white, in comparison to those without COPD. In terms of smoking history, a higher proportion of smokers were found in the COPD group (78.4% vs 21.6%, *P* < 0.001). The COPD group had a higher proportion of individuals with a BMI of 30 or higher. For a comprehensive overview, refer to Table 1.

**TABLE 1:** Patient demographics and baseline characteristics.

Characteristic	COPD			*P*-value
	**Overall, *N* = 2,300**	**No, *N* = 2,017**	**Yes, *N* = 283**	
**Gender**	–	–	–	0.065
Female	1,093 (47.5%)	944 (46.8%)	149 (52.7%)	–
Male	1,207 (52.5%)	1,073 (53.2%)	134 (47.3%)	–
**Age**	–	–	–	< 0.001
> 60	1,100 (47.8%)	924 (45.8%)	176 (62.2%)	–
40–60	1,200 (52.2%)	1,093 (54.2%)	107 (37.8%)	–
**Race**	–	–	–	< 0.001
Mexican American	259 (11.3%)	248 (12.3%)	11 (3.9%)	–
Non-Hispanic black	587 (25.5%)	527 (26.1%)	60 (21.2%)	–
Non-Hispanic white	883 (38.4%)	721 (35.7%)	162 (57.2%)	–
Other Hispanic	247 (10.7%)	224 (11.1%)	23 (8.1%)	–
Other race	324 (14.1%)	297 (14.7%)	27 (9.5%)	–
**Education level**	–	–	–	< 0.001
9–11th grade (includes 12th grade with no diploma)	271 (11.8%)	225 (11.2%)	46 (16.3%)	–
College graduate or above	559 (24.3%)	529 (26.2%)	30 (10.6%)	–
High school graduate/GED or equivalent	566 (24.6%)	469 (23.3%)	97 (34.3%)	–
Less than 9th grade	181 (7.9%)	170 (8.4%)	11 (3.9%)	–
Some college or AA degree	723 (31.4%)	624 (30.9%)	99 (35.0%)	–
**Marital status**	–	–	–	< 0.001
Married/living with partner	1,430 (62.2%)	1,288 (63.9%)	142 (50.2%)	–
Never married	210 (9.1%)	176 (8.7%)	34 (12.0%)	–
Widowed/divorced/separated	660 (28.7%)	553 (27.4%)	107 (37.8%)	–
**Smoking**	–	–	–	< 0.001
No	1,124 (48.9%)	1,063 (52.7%)	61 (21.6%)	–
Yes	1,176 (51.1%)	954 (47.3%)	222 (78.4%)	–
**Asthma**	–	–	–	< 0.001
No	1,933 (84.0%)	1,766 (87.6%)	167 (59.0%)	–
Yes	367 (16.0%)	251 (12.4%)	116 (41.0%)	–
**Congestive heart failure**	–	–	–	< 0.001
No	2,180 (94.8%)	1,939 (96.1%)	241 (85.2%)	–
Yes	120 (5.2%)	78 (3.9%)	42 (14.8%)	–
**Coronary heart disease**	–	–	–	< 0.001
No	2,154 (93.7%)	1,915 (94.9%)	239 (84.5%)	–
Yes	146 (6.3%)	102 (5.1%)	44 (15.5%)	–
**Angina pectoris**	–	–	–	< 0.001
No	2,216 (96.3%)	1,963 (97.3%)	253 (89.4%)	–
Yes	84 (3.7%)	54 (2.7%)	30 (10.6%)	–
**Heart attack**	–	–	–	< 0.001
No	2,147 (93.3%)	1,913 (94.8%)	234 (82.7%)	–
Yes	153 (6.7%)	104 (5.2%)	49 (17.3%)	–
**Liver condition**	–	–	–	0.057
No	2,149 (93.4%)	1,892 (93.8%)	257 (90.8%)	–
Yes	151 (6.6%)	125 (6.2%)	26 (9.2%)	–
**Diabetes**	–	–	–	< 0.001
No	1,800 (78.3%)	1,609 (79.8%)	191 (67.5%)	–
Yes	500 (21.7%)	408 (20.2%)	92 (32.5%)	–
**Drinking**	–	–	–	< 0.001
No	1,904 (82.8%)	1,701 (84.3%)	203 (71.7%)	–
Yes	396 (17.2%)	316 (15.7%)	80 (28.3%)	–
**Body mass index (kg/m** ^2^ **)**	–	–	–	0.002
< 25	498 (21.7%)	445 (22.1%)	53 (18.7%)	–
≥ 30	1,000 (43.5%)	849 (42.1%)	151 (53.4%)	–
25–30	802 (34.9%)	723 (35.8%)	79 (27.9%)	–
**CLR**	–	–	–	< 0.001
Q1 (< –0.66)	573 (24.9%)	525 (26.0%)	48 (17.0%)	–
Q2 (–0.66 to 0.15)	573 (24.9%)	525 (26.0%)	48 (17.0%)	–
Q3 (0.15–0.90)	577 (25.1%)	497 (24.6%)	80 (28.3%)	–
Q4 (≥ 0.90)	575 (25.0%)	469 (23.3%)	106 (37.5%)	–
**CALLY index**	–	–	–	< 0.001
Q1 (< 0.48)	575 (25.0%)	467 (23.2%)	108 (38.2%)	–
Q2 (0.48–1.23)	575 (25.0%)	526 (26.1%)	49 (17.3%)	–
Q3 (1.23–2.05)	577 (25.1%)	497 (24.6%)	80 (28.3%)	–
Q4 (≥ 2.05)	575 (25.0%)	469 (23.3%)	106 (37.5%)	–
**NLR**	–	–	–	< 0.001
Q1 (< 1.44)	575 (25.0%)	523 (25.9%)	52 (18.4%)	–
Q2 (1.44–1.96)	573 (24.9%)	515 (25.5%)	58 (20.5%)	–
Q3 (1.96–2.67)	573 (24.9%)	497 (24.6%)	76 (26.9%)	–
Q4 (≥ 2.67)	579 (25.2%)	482 (23.9%)	97 (34.3%)	–
**Albumin (g/dL)**	4.00 (3.80, 4.20)	4.00 (3.80, 4.20)	3.90 (3.70, 4.10)	< 0.001
**Fasting glucose (mg/dL)**	107 (98, 121)	106 (98, 120)	109 (100, 127)	0.019
**HS C-reactive protein (mg/L)**	2.2 (1.0, 4.5)	2.0 (0.9, 4.2)	3.5 (1.5, 6.1)	< 0.001
**White blood cell (1,000 cells/μL)**	6.45 (5.30, 7.80)	6.30 (5.20, 7.70)	7.30 (6.05, 8.65)	< 0.001
**Lymphocyte (1,000 cells/μL)**	1.90 (1.50, 2.30)	1.90 (1.50, 2.30)	1.90 (1.50, 2.50)	0.366
**Monocyte (1,000 cells/μL)**	0.50 (0.40, 0.60)	0.50 (0.40, 0.60)	0.60 (0.50, 0.75)	< 0.001
**Red blood cell (million cells/μL)**	4.72 (4.40, 5.04)	4.72 (4.40, 5.05)	4.71 (4.40, 4.99)	0.565
**Hemoglobin (g/dL)**	14.20 (13.20, 15.20)	14.20 (13.20, 15.20)	14.10 (13.05, 15.20)	0.481
**Red cell distribution width (%)**	13.70 (13.20, 14.40)	13.60 (13.20, 14.30)	14.10 (13.40, 14.90)	< 0.001
**Triglyceride (mg/dL)**	97 (67, 140)	97 (67, 138)	103 (72, 151)	0.031

### 3.2 Correlation among the study variables and COPD

Table 2 presents the outcomes of univariate and multivariate logistic regression analyses, with the latter examining potential associations between study variables and COPD. In Model 1, without covariates, the CALLY index showed a strong negative association with COPD prevalence (OR = 0.73, 95% CI 0.66–0.81, *P* < 0.0001), while increases in CLR and NLR were significantly linked to higher COPD risk (CLR: OR = 1.36, 95% CI 1.22–1.51, *P* < 0.0001; NLR: OR = 1.23, 95% CI 1.13–1.34, *P* < 0.0001). After adjustment for sex, age, and race in Model 2, the strong negative association remained significant (OR = 74, 95% CI 0.66–0.82, *P* < 0.0001), and the associations of CLR and NLR with COPD risk were consistent with those shown in Model 1. Model 3, fully adjusted for potential confounding variables, showed that the prevalence of COPD increased by 19% for each unit decrease in the CALLY index (OR = 0.81, 95% CI 0.71–0.92, *P* < 0.0001). A unit increase in either CLR or NLR is associated with an increase in COPD prevalence. Specifically, each unit increase in CLR is linked to a 23% rise in COPD prevalence (OR = 1.23, 95% CI 1.08–1.40, *P* < 0.0001), while each unit increase in NLR is associated with a 13% increase in COPD prevalence (OR = 1.13, 95% CI 1.01–1.27, *P* < 0.0001).

**TABLE 2:** Multivariable analysis on the associations between CALLY index, CLR, NLR, white blood cell, monocyte, albumin and COPD.

Characteristic	OR (95% CI); *P*-value		
	Model 1	Model 2	Model 3
**CALLY index**	**0.73 (0.66, 0.81); < 0.001**	**0.74 (0.66, 0.82); < 0.001**	**0.81 (0.71, 0.92); 0.001**
Q1 (< 0.48)	Reference	Reference	Reference
Q2 (0.48–1.23)	**0.66 (0.48, 0.90); 0.010**	**0.63 (0.45, 0.87); 0.005**	0.69 (0.48, 1.01); 0.054
Q3 (1.23–2.05)	**0.46 (0.32, 0.64); < 0.001**	**0.47 (0.32, 0.67); < 0.001**	**0.57 (0.38, 0.86); 0.007**
Q4 (≥ 2.05)	**0.36 (0.24, 0.52); < 0.001**	**0.36 (0.25, 0.53); < 0.001**	**0.49 (0.31, 0.76); 0.002**
**CLR**	**1.36 (1.22, 1.51); < 0.001**	**1.34 (1.20, 1.50); < 0.001**	**1.23 (1.08, 1.40); < 0.001**
Q1 (< –0.66)	Reference	Reference	Reference
Q2 (–0.66 to 0.15)	0.98 (0.65, 1.49); 0.930	0.97 (0.64, 1.49); 0.903	0.92 (0.58, 1.48); 0.744
Q3 (0.15–0.90)	**1.73 (1.19, 2.53); 0.004**	**1.62 (1.11, 2.40); 0.014**	1.33 (0.87, 2.05); 0.194
Q4 (≥ 0.90)	**2.43 (1.70, 3.50); < 0.001**	**2.36 (1.64, 3.46); < 0.001**	**1.79 (1.17, 2.77); 0.008**
**NLR**	**1.23 (1.13, 1.34); < 0.001**	**1.16 (1.06, 1.26); 0.002**	**1.13 (1.01, 1.27); 0.038**
Q1 (< 1.44)	Reference	Reference	Reference
Q2 (1.44–1.96)	1.13 (0.76, 1.68); 0.535	1.03 (0.69, 1.55); 0.868	1.18 (0.75, 1.88); 0.477
Q3 (1.96–2.67)	**1.53 (1.05, 2.23); 0.027**	1.30 (0.88, 1.92); 0.183	1.14 (0.72, 1.81); 0.583
Q4 (≥ 2.67)	**2.03 (1.43, 2.92); < 0.001**	**1.54 (1.06, 2.26); 0.025**	1.34 (0.83, 2.19); 0.234
**White blood cell**	**1.21 (1.14, 1.27); < 0.001**	**1.21 (1.14, 1.28); < 0.001**	**1.08 (1.00, 1.17); 0.045**
**Monocyte**	**8.65 (4.81, 15.68); < 0.001**	**7.83 (4.27, 14.54); < 0.001**	**2.87 (1.40, 5.87); 0.004**

*P*-value < 0.05 is shown in bold. Model 1: no covariates were adjusted. Model 2: adjusted for age, gender, race. Model 3: adjusted for age, gender, race, education level, marital status, smoking, asthma, heart attack, congestive heart failure, coronary heart disease, angina pectoris, liver condition, diabetes, drinking, red blood cell count, hemoglobin, red cell distribution width, lymphocyte number, FBG. In the sensitivity analysis, the CALLY index, CLR, and NLR were converted from a continuous variable to a categorical variable (quartile). OR, odds ratio; CI, confidence interval; COPD, chronic obstructive pulmonary disease; CALLY index, CRP-albumin-lymphocyte index; CLR, C-reactive protein to lymphocyte ratio; NLR, neutrophil-to-lymphocyte ratio; FBG, fasting glucose.

In the sensitivity analyses, the three inflammation indicators were categorized into quartiles, using the first quartile as the reference group. Model 3 revealed that participants in the top quartile of the CALLY index exhibited a 51% reduced prevalence of COPD compared to those in the bottom quartile, with an odds ratio of 0.49 (95% CI 0.31–0.76, *P* < 0.0001). This implies that a higher CALLY index might be a protective factor and is associated with a lower risk of developing COPD. Regarding the CLR index, the study found that compared with the participants in the lowest quartile (Q1), those in the highest quartile (Q4) had a significantly higher risk of developing COPD. This indicates that the higher the CLR is, the greater the likelihood of developing COPD.

### 3.3 Stratified analysis

**FIGURE 2:**
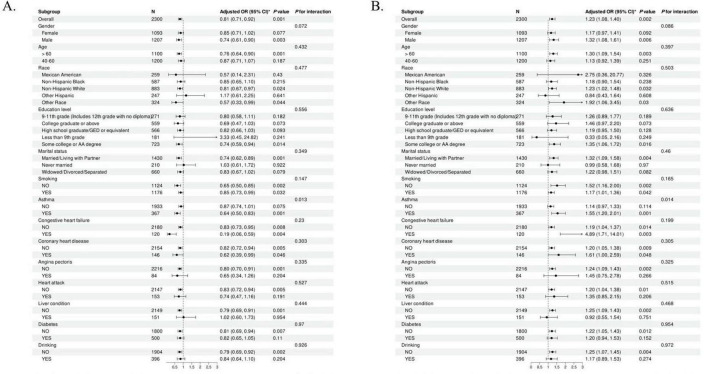
Subgroup analysis examining the association between the Inflammation indicators and COPD. **(A)** The subgroup analysis of COPD and CALLY index. **(B)** The subgroup analysis of COPD and CLR. CALLY index, C-reactive protein-albumin-lymphocyte index; CLR, C-reactive protein to lymphocyte ratio.

Stratified analyses were performed to assess the extent to which the multiple regression outcomes accurately depicted the relationship between the CALLY index and CLR—both indicators of inflammation—and COPD across different subgroups. The results of this analysis are presented in Figure 2.

A subgroup analysis investigated the connection between the CALLY index and COPD. The negative association between the CALLY Index and COPD was stronger in males over 60 and non-Hispanic whites. This phenomenon was also evident among those who were married or cohabiting and had congestive heart failure. The positive association between CLR and COPD was confirmed in the same subgroups. In addition, the *P*-values for the interactions in all subgroups were greater than 0.05, suggesting the stability and consistency of our findings across subgroups.

### 3.4 Correlation between inflammation indicators and COPD

**FIGURE 3:**
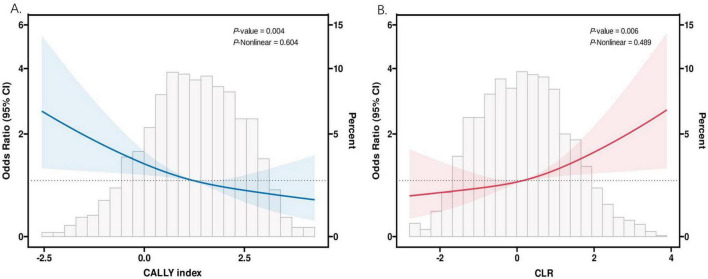
The association between CALLY index and CLR and COPD. **(A)** The RCS analysis of COPD and CALLY index. **(B)** The RCS analysis of COPD and CLR. CALLY index, C-reactive protein-albumin-lymphocyte index; CLR, C-reactive protein to lymphocyte ratio.

Restricted cubic spline modeling was employed to analyze the dose-response relationship between the two inflammatory indicators and COPD. Figure 3 illustrates that the adjusted smoothed curves, considering all covariates, indicate a negative correlation between the CALLY index and COPD (*P*-non-linear = 0.604), while the CLR exhibits a positive correlation with COPD (*P*-non-linear = 0.489). The study confirms that there is an opposing linear relationship between these two novel inflammatory indices and COPD.

### 3.5 ROC analysis outcomes

**FIGURE 4:**
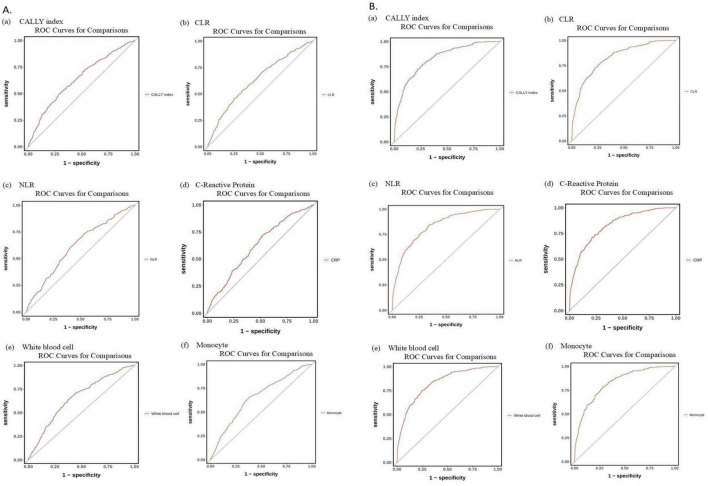
**(A)** ROC curves of (a) C-reactive protein, albumin, and lymphocyte (CALLY) index, (b) C-reactive protein to lymphocyte ratio (CLR), (c) neutrophil-to lymphocyte ratio (NLR), (d) C-reactive protein, (e) White blood cell, and (f) Monocyte in predicting the risk of chronic obstructive pulmonary disease (COPD) (age-adjusted). **(B)** ROC curves for predicting COPD risk, adjusted for significant variables from Supplementary Table 1, using: (a) CALLY index, (b) CLR, (c) NLR, (d) C-reactive protein, (e) White blood cell count, and (f) Monocyte levels. ROC, receiver operating characteristics curve.

Figure 4 and Table 3 display the ROC analyses for predicting COPD risk using the CALLY index, CLR, NLR, CRP, white blood cells, and monocytes.

**TABLE 3:** Receiver operating characteristics curve (ROC) analysis of C-reactive protein, albumin, and lymphocyte (CALLY) index, C-reactive protein to lymphocyte ratio (CLR), neutrophil-to lymphocyte ratio (NLR), leukocytes, monocytes, and C-reactive protein in predicting the risk of chronic obstructive pulmonary disease (COPD) onset in a middle-aged and older population in the United States.

Characteristic	AUC	95% CI
**Model 1**		
CALLY index	0.637	(0.602–0.672)
CLR	0.634	(0.599–0.669)
NLR	0.619	(0.585–0.653)
C-reactive protein	0.620	(0.587–0.654)
White blood cell	0.659	(0.626–0.692)
Monocyte	0.657	(0.623–0.691)
**Model 2**		
CALLY index	0.831	(0.806–0.856)
CLR	0.831	(0.806–0.855)
NLR	0.830	(0.805–0.855)
C-reactive protein	0.829	(0.804–0.854)
White blood cell	0.830	(0.805–0.854)
Monocyte	0.830	(0.806–0.855)

Model 1: adjusted for age (continuous). Model 2: adjusted for the variables that were significant in Supplementary Table 1 in the Supporting Information, including age (continuous), education level, race, smoking, asthma, fasting glucose, lymphocyte, red blood cell, red cell distribution width, history of coronary heart disease, angina pectoris, heart attack, diabetes and drinking.

The age-adjusted AUC (Model 1) was 0.637 (95% CI: 0.602–0.672) for CALLY index, 0.634 (95% CI: 0.599–0.669) for CLR, 0.619 (95% CI: 0.585–0.653) for NLR, and 0.620 (95% CI: 0.587–0.654) for CRP. White blood cells were 0.659 (95% CI: 0.626–0.692), and monocytes were 0.657 (95% CI: 0.623–0.691).

Following additional adjustments for significant COPD risk factors (Model 2), the AUC values were as follows: CALLY index at 0.831 (95% CI 0.806–0.856), CLR at 0.831 (95% CI 0.806–0.855), NLR at 0.830 (95% CI 0.805–0.855), and CRP at 0.829 (95% CI 0.804–0.854). White blood cells and monocytes both had a value of 0.830, with 95% confidence intervals of 0.805–0.854 and 0.806–0.855, respectively (refer to Figures 4A, B and Table 3).

## 4 Discussion

No academic studies in the United States have examined the correlation between the CALLY Index, CLR, and COPD in middle-aged and older adults. Researchers sampled 15,560 people between 2017 and March 2020, with a final 2,300 enrolled after multiple screenings to determine if the inflammatory index was associated with COPD. Multifactorial logistic regression analyses revealed a strong negative association between the CALLY index and COPD prevalence, while CLR exhibited a positive relationship with COPD. Additional subgroup analyses were performed to assess the precision and robustness of this association. Figure 2 presents the results. The inverse relationship between the CALLY index and COPD was stronger among participants aged 60 and above, as well as non-Hispanic whites. The opposite conclusion can be affirmed by subgroup analyses of the CLR. Furthermore, this phenomenon was consistent across the entire population sample. An RCS curve-fitting model was constructed to further assess the reliability of the results.

The study reveals a substantial finding that the CALLY index is negatively correlated with COPD, with a *P*-non-linearity value of 0.604, indicating that a lower CALLY Index may increase COPD incidence. Consistent with previous descriptions, the positive association of CLR with COPD was also confirmed here. After adjusting for relevant variables, ROC curve analysis revealed that the CALLY index and CLR are more predictive of COPD risk compared to other hematological inflammation indicators.

The prevalence of COPD among individuals with obesity is exhibiting a year-on-year increase (20). COPD involves ongoing airflow restriction linked to an increased chronic inflammatory response in the respiratory system. Patients with COPD often experience pulmonary alterations accompanied by changes in the composition of their body (21, 22), dysfunction of the skeletal musculature (23), diseases related to the cardiovascular and circulatory systems (24), mood disorder (25), inflammatory bone disease, decreased physiological tolerance of exercise (26), and inflammation throughout the body (27). Inflammatory responses could constitute a significant risk factor for additional complications associated with COPD (28). COPD patients face a heightened risk of extrapulmonary manifestations and comorbidities, including cardiovascular disease and pulmonary carcinoma, which substantially assist in the transmission of diseases (29–31). The assessment of lung function is presently regarded as the primary diagnostic approach for COPD. However, lung function testing is only performed when respiratory symptoms occur (7, 8). A United States study indicates that despite improvements in spirometry use for COPD detection and management in primary care following the NLHEP implementation, 70% of COPD patients have not received spirometry testing (9). A straightforward biological marker for early-stage COPD screening could aid in its evaluation, management, and treatment.

As one of the most widely studied biomarkers in COPD, CRP occurs during pathological changes (32–34), severe destruction of lung parenchyma, and the onset of inflammatory response in this disease (35). A previous study elucidated that immediate detection of CRP can reduce the unnecessary use of antibiotics for patients with acute exacerbations of COPD and help improve antibiotic management to reduce overtreatment (36). In addition to identifying better COPD prevention and treatment strategies, aggressive or supportive treatment for COPD can also be facilitated through the pursuit of novel markers (37–40). Wang’s team investigated the link between NLR and lung function decline, indicating COPD risk. The study indicates that the NLR is a significant biomarker linked to impaired lung function and increased COPD risk, based on its correlation with DNA methylation profiles, by examining the clinical features of patients with acute COPD exacerbations and the NLR’s prognostic value. The NLR is acknowledged as an effective prognostic biomarker for severe acute COPD exacerbation due to its proven performance in various studies and its accessibility (41).

The CALLY index is an innovative measure that integrates various aspects of nutritional status, immune function, and inflammation levels (42–45). Acknowledging the TNM staging system’s limitations, including tumor heterogeneity, molecular traits, and immune status, Liu’s team developed and validated the CALLY index for predicting non-small cell lung cancer prognosis. They also created a CALLY-based nomogram model to forecast long-term outcomes for NSCLC patients. The study demonstrated that compared to high scores, low CALLY index scores were associated with much poorer overall survival, aligning with our findings in COPD. The new predictive model surpasses the traditional TNM staging system by simultaneously integrating multiple independent variables to numerically estimate the likelihood of clinical events. In addition, based on the morphometric map of the CALLY index, clinicians can risk-stratify each lung cancer patient according to different risk factors and thus develop personalized treatment strategies as early as possible (12). A recent study by Nagano et al. (14) assessed the prognostic significance of preoperative inflammatory markers, the findings of this study indicate that, among the inflammatory markers analyzed, the CLR demonstrates highly significant predictive advantages in forecasting surgical outcomes for those diagnosed with non-small cell lung cancer. This marker can serve as a critical reference for healthcare professionals in assessing postoperative recovery and the risk of disease progression, thereby facilitating the development of more personalized treatment and follow-up plans.

No research has explored the association between the CALLY index and CLR, both indicators of inflammation, and COPD. This survey is the first cross-sectional study to examine their relationship with COPD. The survey used NHANES data from 2017 to March 2020, with 15,560 participants taking part in the 2017–2020/3 survey cycle. A final sample size of 2,300 was achieved by applying strict inclusion and exclusion criteria to ensure comprehensive and accurate results. In this study, we confirmed that both the CALLY index and CLR predicted the risk of developing COPD, and both performed slightly better than the NLR.

This study has multiple advantages. The study utilized a sample that is representative of middle-aged and older Americans, balanced for age and gender, to evaluate the relationship between the CALLY Index, CLR, and COPD, ensuring standardized and high-quality measurements reflective of the general United States population. This study uniquely explores the association between these two novel inflammatory indices and COPD. Second, we adjusted for potential confounders by adjusting for covariates such as sociodemographic factors and lifestyle variables. Third, we performed subgroup analyses to elucidate their respective differential effects on COPD in different subgroups. Through fully adjusted models and RCS curve fitting, we identified a linear negative association between the CALLY index and COPD in middle-aged and older Americans, contrasting with the positive correlation observed between CLR and COPD, thereby minimizing potential confounding bias. After adjusting for all relevant variables, the CALLY index and CLR were found to be more effective in predicting the risk of developing COPD, with an improved AUC of 0.831. These findings suggest that, among other markers of hematological inflammation, the CALLY index and CLR may be better indicators for estimating the probability of the risk of developing COPD in such patients. The team led by Karakayali et al. (46) found that the HALP (hemoglobin, albumin, lymphocyte, and platelet) score, as an indicator reflecting nutritional status and systemic inflammation, is associated with the in-hospital mortality of patients with ST-segment elevation myocardial infarction. This is similar to the conclusion of our study, in which the CALLY index and CLR reflect the body’s inflammatory and nutritional status and thus affect the disease risk, further supporting the importance of inflammation - and nutrition-related indicators in the assessment of disease prognosis.

The CALLY index integrates serum albumin, lymphocyte count, and CRP, enabling it to comprehensively reflect an individual’s nutritional status, immune function, and inflammation level (12). A higher CALLY index implies a better nutritional status (normal albumin level), stronger immune function (adequate lymphocyte count), and a lower inflammation level (low CRP level). These factors may act together to reduce the risk of COPD, thus giving it good predictive ability for COPD risk (36, 47).

An elevated CRP level indicates the presence of an inflammatory response in the body. When the lymphocyte count is relatively low, the CLR value increases, suggesting a relatively strong inflammatory response and a relatively weak immune defense (14). This makes it difficult for the body to effectively deal with inflammation, further exacerbating the pathological damage in the lungs and increasing the risk of COPD, making it an effective predictor of COPD risk.

During the development of COPD, there is a disorder in the immune function, with abnormalities in the number or function of lymphocytes. When the CALLY index is high, the number of lymphocytes is sufficient, and the immune function is relatively normal, which can better regulate the immune response, resist pathogen infections, reduce lung inflammation, and lower the risk of COPD. Conversely, when the CLR increases, the number of lymphocytes is relatively insufficient, the immune-regulatory function is impaired, and the inflammatory response cannot be effectively controlled, thus promoting the development of COPD.

The pathogenesis of COPD is closely related to multiple inflammatory signaling pathways, such as the nuclear factor - κB (NF - κB) pathway (37, 48). Under normal circumstances, NF - κB is in an inhibitory state. When stimulated by inflammation, NF - κB is activated, translocates into the nucleus, and initiates the expression of a series of inflammation-related genes, including pro-inflammatory cytokines (such as tumor necrosis factor - α, interleukin - 6, etc.,) and chemokines, leading to an exacerbation of the inflammatory response (49). CRP can promote the inflammatory response by activating the NF - κB pathway. Albumin in the CALLY index may have antioxidant and anti-inflammatory effects, which can inhibit the activation of the NF - κB pathway and reduce the production of inflammatory factors (50). Therefore, when the CALLY index is high, the activation of the inflammatory signaling pathway is inhibited, which helps to reduce the risk of COPD. When the CLR increases, it means that the relative amount of CRP to lymphocytes increases, which may further activate the NF - κB pathway, aggravate lung inflammation, and increase the risk of COPD (51).

Oxidative stress also plays an important role in the pathogenesis of COPD. A large number of reactive oxygen species (ROS) generated by oxidative stress can damage airway epithelial cells, lung parenchymal cells, etc., leading to cell dysfunction, aggravated inflammation, and tissue damage (52). Lymphocytes have a certain antioxidant capacity and can scavenge ROS by producing antioxidant enzymes (such as superoxide dismutase, glutathione peroxidase, etc.) (53). When the CALLY index is high, the number of lymphocytes is sufficient, and the antioxidant capacity is strong, which can effectively reduce the damage of oxidative stress to lung tissues and lower the risk of COPD (54). When the CLR increases, the number of lymphocytes is relatively insufficient, the oxidative stress damage cannot be effectively repaired, the inflammatory response persists, and thus promoting the development of COPD (55).

A significant limitation of our study was its cross-sectional design, which restricted the ability to determine causal and temporal relationships between the CALLY index, CLR, and COPD. Even though multiple covariates such as age, gender, race, education level, marital status, smoking history, drinking history, and diabetes were included in the analysis, there may still be other unmeasured factors influencing the relationship between COPD risk and inflammatory markers. For example, environmental factors such as long-term exposure to air pollution and occupational dust may simultaneously affect the occurrence of COPD and the levels of inflammatory markers, but these were not measured and adjusted for in this study. Individual genetic susceptibility may also play an important role in the development of COPD and is related to the inflammatory response. However, this information is missing from the study. These unmeasured factors may interfere with the observed association between inflammatory markers and COPD risk in the study. In addition, the association between these two inflammatory indices and COPD needs to be further validated by larger cohort studies and more prospective studies. Even after additional covariates are screened and employed to control for detection bias, unexplained confounding factors may persist. Although these factors might contribute to COPD etiology, they are not explicitly documented in the NHANES database. As our research primarily targeted middle-aged and elderly individuals, we excluded COPD results for younger participants.

## 5 Conclusion

In conclusion, our study identified a linear negative relationship between the CALLY index and COPD, independent of potential confounders. A higher CLR is linked to an elevated risk of COPD development. Both the CALLY index and CLR were superior in predicting the risk of developing COPD. Our findings emphasize that the CALLY index with CLR may be a new inflammatory early warning biomarker for COPD. The inherent limitations of studies using cross-sectional methods necessitate further prospective studies to verify the causality of this association.

## Data Availability

The datasets presented in this study can be found in online repositories. The names of the repository/repositories and accession number(s) can be found below: the datasets for this study were sourced from NHANES (https://www.cdc.gov/nchs/nhanes/).

## Footnotes

1
https://wwwn.cdc.gov/Nchs/Nhanes/2017-2018/P_DEMO.htm#DMDEDUC2atoryProcedureManual
