# Supplementary material for: Association of CALLY index and CLR with COPD risk in middle-aged and older Americans: evidence from NHANES 2017–2020
Source: Front Med (Lausanne). 2025 Apr 17;12:1535415. doi: 10.3389/fmed.2025.1535415 (PMC12043464; doi:10.3389/fmed.2025.1535415)
Supplement: Supplementary file 1 [file Table_1.docx]

Table S1. Univariate analysis of associations between study variables and COPD

|  | COPD | |
| --- | --- | --- |
| Variables | OR (95% CI) | *p*-value |
| **Age** | **1.03 (1.02-1.04)** | **<0.001** |
| **Gender** |  | |
| Female | Reference |  |
| Male | 0.79 (0.62-1.01) | 0.065 |
| **Race** |  |  |
| Mexican American | Reference |  |
| Non-Hispanic Black | **2.57 (1.38-5.23)** | **0.005** |
| Non-Hispanic White | **5.07 (2.83-10.05)** | **<0.001** |
| Other Hispanic | **2.31 (1.13-5.04)** | **0.026** |
| Other Race | 2.05 (1.02-4.39) | 0.051 |
| **Education level** |  | |
| 9-11th grade (Includes 12th grade with no diploma) | Reference |  |
| College graduate or above | **0.28 (0.17-0.45)** | **<0.001** |
| High school graduate/GED or equivalent | 1.01 (0.69-1.50) | 0.953 |
| Less than 9th grade | **0.32 (0.15-0.61)** | **0.001** |
| Some college or AA degree | 0.78 (0.53-1.14) | 0.193 |
| **Marital status** |  | |
| Married/Living with Partner | Reference |  |
| Never married | **1.75 (1.15-2.60)** | **0.007** |
| Widowed/Divorced/Separated | **1.76 (1.34-2.30)** | **<0.001** |
| **Smoking** |  |  |
| No | Reference |  |
| YES | **4.06 (3.03-5.50)** | **<0.001** |
| **Asthma** |  | |
| No | Reference |  |
| YES | **4.89 (3.72-6.41)** | **<0.001** |
| **Congestive heart failure** |  | |
| NO | Reference |  |
| YES | **4.33 (2.89-6.42)** | **<0.001** |
| **Coronary heart disease** |  | |
| NO | Reference |  |
| YES | **3.46 (2.35-5.01)** | **<0.001** |
| **Angina pectoris** |  | |
| NO | Reference |  |
| YES | **4.31 (2.68-6.82)** | **<0.001** |
| **Heart attack** |  | |
| NO | Reference |  |
| YES | **3.85 (2.65-5.53)** | **<0.001** |
| **Liver condition** |  | |
| NO | Reference |  |
| YES | 1.53 (0.97-2.34) | 0.059 |
| **Diabetes** |  | |
| NO | Reference |  |
| YES | **1.90 (1.44-2.49)** | **<0.001** |
| **Drinking** |  | |
| NO | Reference |  |
| YES | **2.12 (1.59-2.81)** | **<0.001** |
| **Fasting Glucose** | **1.00 (1.00-1.01)** | **0.011** |
| **Lymphocyte** | **1.18 (1.05-1.35)** | **0.011** |
| **Red blood cell** | 0.90 (0.71-1.15) | 0.413 |
| **Hemoglobin** | 0.97 (0.89-1.04) | 0.378 |
| **Red cell distribution width** | **1.22 (1.12-1.31)** | **<0.001** |

HR, hazard ratio; CI, confidence interval; COPD, chronic obstructive pulmonary disease.

*P*-value < 0.05 is shown in bold.
